# DSM 5 through several lenses: how eating disorders can look different

**DOI:** 10.1186/2050-2974-3-S1-O53

**Published:** 2015-11-23

**Authors:** Anthea Fursland, David Erceg-Hurn, Susan Byrne

**Affiliations:** 1 Centre for Clinical Interventions, Perth, Australia; 2 University of Western Australia, Perth, Australia

## 

The Eating Disorder Examination (EDE), a structured diagnostic interview, is the ‘gold standard’ for diagnosing eating disorders (EDs). There are well established algorithms for DSM-IV diagnosis but not for DSM-5. The various algorithms proposed for DSM-5 differ in terms of their use of the EDE to operationalise diagnostic criteria such as low weight, episodes of binge eating, and compensatory behaviours to prevent weight gain. We compared four DSM-5 EDE diagnostic algorithms, to evaluate the extent to which the choice of algorithm affects the diagnosis given to a patient. Participants were over 400 patients referred to a specialist West Australian ED outpatient clinic. Clinical psychologists administered the EDE to each patient and these EDE scores were analysed using four different algorithms. We found that, for a significant minority of patients, the diagnosis derived from the EDE changed depending on what diagnostic algorithm was used. The results indicate that competing algorithms for diagnosing DSM-5 EDs using the EDE are not interchangeable, and that the choice of algorithm has a practical impact on which diagnosis is given. The significant implications of these findings for research and clinical practice will be discussed.

